# Unveiling the genetic relationship between Alzheimer’s disease and chronic pain‐related disorders

**DOI:** 10.1002/alz70861_108281

**Published:** 2025-12-23

**Authors:** Olasunkanmi David Bamidele, Ayeisha Milligan Armstrong, Tenielle Porter, Eleanor K. O’Brien, Giuseppe Verdile, Emmanuel O Adewuyi, Simon M. Laws, ADOPIC Research Consortium

**Affiliations:** ^1^ Faculty of Pharmaceutical Sciences, University of Ilorin, Ilorin, Kwara State Nigeria; ^2^ Centre for Precision Health, Edith Cowan University, Joondalup, Western Australia Australia; ^3^ Curtin Medical Research Institute, Curtin University, Perth, Western Australia Australia; ^4^ Curtin Medical School, Curtin University, Bentley, Western Australia Australia; ^5^ Collaborative Genomics and Translation Group, School of Medical and Health Sciences, Edith Cowan University, Joondalup, Western Australia Australia; ^6^ School of Medical and Health Sciences, Edith Cowan University, Joondalup, Western Australia Australia; ^7^ Collaborative Genomics and Translation Group, Edith Cowan University, Joondalup, Western Australia Australia

## Abstract

**Background:**

Observational studies suggest an association between Alzheimer’s disease (AD) and chronic pain‐related disorders (CPDs). Inflammatory mechanisms may underpin this link, but specific pathways remain unclear. Investigating shared genetic factors may provide valuable insights.

**Method:**

We used large‐scale, publicly available genome‐wide association study (GWAS) summary statistics to assess the genetic relationship between AD and CPDs. Linkage disequilibrium score regression (LDSC) and two‐sample Mendelian randomisation (2SMR) analyses were conducted to examine genetic correlation and causal effects. We also analysed genome‐wide overlap and identified shared genes. In a longitudinal cohort, these genes were evaluated against brain‐health phenotypes, including cognition, brain volumetrics, and PET‐derived estimates of the onset of high brain amyloid‐beta (Aβ) burden.

**Result:**

LDSC estimates reveal positive and significant genetic correlation (*r*_G_) of AD with dorsopathy (*r*_G_ = 0.09, *p* = 3.31 x 10^‐4^); multisite chronic pain (MCP) (*r*_G_ = 0.26, *p* = 5.43 x 10^‐10^); NSAIDs use (*r*_G_ = 0.10, *p* = 6.70 x 10^‐3^); headache (*r*_G_ = 0.15, *p* = 3.19 x 10^‐5^), and osteoarthritis (*r*_G_ = 0.18, *p* = 3.23 x 10^‐5^), but not ankylosis or rheumatoid arthritis. We observed a highly significant causal effect of MCP on AD [IVW (OR = 1.12, 95%CI = 1.06‐1.18, *p* = 7.14 x 10^‐5^)]. AD showed risk‐decreasing effects on ankylosis [IVW (OR = 0.67, 95%CI = 0.46‐0.95, *p* = 2.68 x 10^‐2^) and dorsopathy [IVW (OR = 0.90, 95%CI = 0.84‐0.97, *p* = 4.07 x 10^‐3^)]. Gene overlap analyses showed significant overlap between AD and multiple CPDs. Several genes were associated with at least one brain‐health‐related phenotype.

**Conclusion:**

Our study reveals a significant genetic correlation between AD and CPDs, except for ankylosis and rheumatoid arthritis, and the causal effects of MCP on AD. It identified significant genetic overlap and shared genes by AD across the CPDs, implicating shared biological mechanisms.

